# Supplementary material for: Development of a virtual classroom for pre-analytical phase of laboratory medicine for undergraduate medical students using the Delphi technique
Source: PLoS One. 2022 Apr 6;17(4):e0264447. doi: 10.1371/journal.pone.0264447 (PMC8985926; doi:10.1371/journal.pone.0264447)
Supplement: Attachment — Submitted filename: Response to reviewers.docx [file pone.0264447.s001.docx]

We've checked your submission and before we can proceed, we need you to address the following issues:

1. We note you state consent was obtained from the participants of this study. However, you have not clarified the type of consent provided. Before we can proceed, please address the following prompts:

a) Did participants provide their written or verbal informed consent to participate in this study?
b) If consent was verbal, please explain i) why written consent was not obtained, ii) how you documented participant consent, and iii) whether the ethics committees/IRB approved this consent procedure.

Response to reviewers: Details about the written consent obtained from the participants was included in the manuscript in the methods section. “A written consent was obtained from all the panel members prior to their participation in the Delphi process. The consent was obtained via email which outlined the Delphi process, expectations from the panel members, and how the data will be used afterwards.” (Page 4; Line 35)
