# Supplementary material for: Development of a virtual classroom for pre-analytical phase of laboratory medicine for undergraduate medical students using the Delphi technique
Source: PLoS One. 2022 Apr 6;17(4):e0264447. doi: 10.1371/journal.pone.0264447 (PMC8985926; doi:10.1371/journal.pone.0264447)
Supplement: Attachment — Submitted filename: Response to Reviewers.docx [file pone.0264447.s002.docx]

1/27/2022

Vineet Kumar Rai, PhD

Academic Editor

PLOS ONE

**Subject: Rebuttal Letter-'Response to Reviewers': PONE-D-21-24285R1; Development of a Virtual Classroom for Pre-Analytical Phase of Laboratory Medicine for Undergraduate Medical Students Using the Delphi Technique**

Dear Editor,

Please find point by point response to all comments and suggestions made by reviewers.

- Journal Requirements: Please review your reference list to ensure that it is complete and correct. If you have cited papers that have been retracted, please include the rationale for doing so in the manuscript text, or remove these references and replace them with relevant current references. Any changes to the reference list should be mentioned in the rebuttal letter that accompanies your revised manuscript. If you need to cite a retracted article, indicate the article’s retracted status in the References list and also include a citation and full reference for the retraction notice. [Note: HTML markup is below. Please do not edit.]

Author’s Response: All references have been verified.

- Reviewers' comments: Reviewer's Responses to Questions

**Comments to the Author**

1. If the authors have adequately addressed your comments raised in a previous round of review and you feel that this manuscript is now acceptable for publication, you may indicate that here to bypass the “Comments to the Author” section, enter your conflict of interest statement in the “Confidential to Editor” section, and submit your "Accept" recommendation.

Reviewer #1: (No Response)

Reviewer #2: All comments have been addressed

Author’s Response: Not needed

- 2. Is the manuscript technically sound, and do the data support the conclusions?

Reviewer #1: (No Response)

Reviewer #2: Partly

Author’s Response: Not needed

- 3. Has the statistical analysis been performed appropriately and rigorously?

Reviewer #1: (No Response)

Reviewer #2: N/A

Author’s Response: Not needed

- 4. Have the authors made all data underlying the findings in their manuscript fully available?

  Reviewer #1: (No Response)

Reviewer #2: Yes

Author’s Response: Not needed

- 5. Is the manuscript presented in an intelligible fashion and written in standard English?

  Reviewer #1: (No Response)\

Reviewer #2: Yes

Author’s Response: Not needed

- 6. Review Comments to the Author

  Reviewer #1: DEAR Authors, I suggest that the review’s add my initial opinion with the revised submission an itemized, point-by-point response to the comments of the reviewers. Thank you.

Reviewer #2: (No Response)

Author’s Response to Reviewer One:

Dear Reviewer these were the suggestions and comments shared by you

“ 1. We note you state consent was obtained from the participants of this study. However, you have not clarified the type of consent provided. Before we can proceed, please address the following prompts:

a) Did participants provide their written or verbal informed consent to participate in this study?

b) If consent was verbal, please explain i) why written consent was not obtained, ii) how you documented participant consent, and iii) whether the ethics committees/IRB approved this consent procedure.”

We have below itemized point-by point response to your comments made then:

Reviewer comment “ 1. We note you state consent was obtained from the participants of this study. However, you have not clarified the type of consent provided. Before we can proceed, please address the following prompts:

a) Did participants provide their written or verbal informed consent to participate in this study?

Author’s Response: Details about the written consent obtained from the participants was included in the manuscript in the methods section. “A written consent was obtained from all the panel members prior to their participation in the Delphi process. The consent was obtained via email which outlined the Delphi process, expectations from the panel members, and how the data will be used afterwards.” (Page 4; Line 35)

b) If consent was verbal, please explain i) why written consent was not obtained, ii) how you documented participant consent, and iii) whether the ethics committees/IRB approved this consent procedure.”

Author’s Response: We did not take verbal consent. Written informed consent was taken from all participants via email and permission from Ethical Review Committee was taken for written consent.

- 7. PLOS authors have the option to publish the peer review history of their article ([what does this mean?](https://journals.plos.org/plosone/s/editorial-and-peer-review-process#loc-peer-review-history)). If published, this will include your full peer review and any attached files. If you choose “no”, your identity will remain anonymous but your review may still be made public. **Do you want your identity to be public for this peer review?** For information about this choice, including consent withdrawal, please see our [Privacy Policy](https://www.plos.org/privacy-policy).

Reviewer #1: No

Reviewer #2: No

Author’s Response: Not needed

While revising your submission, please upload your figure files to the Preflight Analysis and Conversion Engine (PACE) digital diagnostic tool, <https://pacev2.apexcovantage.com/>. PACE helps ensure that figures meet PLOS requirements. To use PACE, you must first register as a user. Registration is free. Then, login and navigate to the UPLOAD tab, where you will find detailed instructions on how to use the tool. If you encounter any issues or have any questions when using PACE, please email PLOS at [figures@plos.org](mailto:figures@plos.org). Please note that Supporting Information files do not need this step.

Author’s Response: We have uploaded and improved the figure files to the Preflight Analysis and Conversion Engine (PACE) digital diagnostic to ensure that figures meet PLOS requirements

Thank you

Dr Lena Jafri,

MBBS, FCPS, FAIMER fellow Philadelphia

Department of Pathology and Laboratory Medicine

Medical College, Pakistan, The Aga Khan University

Stadium Road, P.O. Box 3500, Karachi 74800, Pakistan
